# The virtual patient as a learning tool: a mixed quantitative qualitative study

**DOI:** 10.1186/s12909-018-1395-8

**Published:** 2018-12-06

**Authors:** Andrés Isaza-Restrepo, María Teresa Gómez, Gary Cifuentes, Arturo Argüello

**Affiliations:** 1 School of Medicine and Health Sciences, Medical and Health Sciences Education Research Group, Universidad del Rosario, Carrera 24 No 63C - 69, Bogotá, DC Colombia 111221; 2 School of Education- Los Andes University, Cra 1 Nº 18A - 12, 111711 Bogotá, Colombia; 3 Invento: Creative Solutions for Health, Wellness & Education Corp, Vancouver, BC Canada

**Keywords:** Clinical reasoning, Problem-based learning, Virtual patients, Simulation, Medical education, Teaching, Learning

## Abstract

**Background:**

The use of simulation in medical education has been widely accepted. There are different types of medical simulators that vary in both accuracy to emulate the real world (fidelity) and cost of development or acquisition. There is significant evidence that supports the use of high-fidelity simulators (i.e. mannequins or dummies) to prepare students for clinical environments, less attention has been given to low-fidelity simulators. This article aims to present evidence regarding the effectiveness of a low-fidelity simulator: Virtual Patient (VP), which develops several interactive computer-based clinical scenarios, seeking to promote an alternative learning environment and the development of necessary medical skills such as clinical reasoning in students of medicine.

**Methods:**

A quasi-experimental study was designed to investigate the results on the development of history taking and clinical reasoning skills in a group of undergraduate medical students, in a course devised under the concepts of constructivism in education, which used the Virtual Patient as the fundamental teaching tool. Results were measured through a mixed, quantitative and qualitative study, triangulating the results of the students’ skills evaluation when facing a clinical case represented by an actor patient before and after the course. Additionally, the description of the students’ and tool’s performance was measured by way of a qualitative study.

**Results:**

The comparison of the students’ skills on the evaluation matrix before-and-after the course evidenced a statistically significant advance (*p* < 0.01) in all aspects (interview, physical exam, clinical judgment, relevance of medical exams, and presentation of case). Students described the VP as an easy-to-use and motivating tool for learning without stress, especially at the beginning of their career. VP allowed them to create logical and structured processes, to be wrong without consequences, and to review and reassess information available. From the professor perspective, it allowed a better follow-up of the students’ learning process and favored reflections on the teaching-learning process.

**Conclusions:**

VP proved to be a valuable and useful tool for the development of clinical reasoning and history taking skills in medical students, as part of a constructivist learning course.

## Background

It has been considered that traditional medical learning scheme described in 1904 by William Halstead as “see one, do one” entails a risk for patients when being exposed to inexperienced trainees. Current evidence suggests that medical error represents one of the leading causes of death [1–3]. Hence, patient safety is recognized around the world as a priority, where “trial-error”-based learning cannot be an option in a real patient [4]. The concept of learning curves in the analysis of the performance of multiple medical procedures has also demonstrated the relevance of practice and repetition during the learning process, with a view to reduce error and achieve better outcomes. On another note, changes undergone by healthcare systems in the past decades have affected the characteristics of medical assistance on a global scale, limiting the possibilities for interaction between students and patients. The restricted opportunities for developing skills in medical practice at patient bedside have resulted in a trend towards observational learning, thus affecting medical education. The features of medical care today pose few opportunities for a reflexive instruction, as well as little time for teaching. These circumstances make it necessary to introduce changes to medical education [5].

Besides that, advances in the knowledge acquired on the learning process from diverse disciplines and evidence obtained by education research have fostered the study, development and evaluation of new teaching strategies. Emergent strategies are based on constructivist concepts, which highlight the importance of the repeated interaction between the learner and the object of learning, and the importance of others in the process of knowledge construction [6]. This implies providing the student with the possibility of undergoing experiences in controlled settings where it is possible to make mistakes, learn from error without risk for patients, and the possibility of timely feedback and repetition. This should promote the development of the clinical skills before putting them at the service of patients.

Simulation in medical education has been developed under this context, understood as the use of techniques that invoke or replicate substantial aspects of the real world and replace or magnify the experiences of patients, and allows for experimenting with them artificially in an interactive and guided manner. Nowadays, a myriad of simulation tools are available: simulated patients, virtual patients, static or interactive simulation dummies, task trainers, computerized screen-based simulators, games, etc. These resources are classified according to their fidelity degree. Their applications include either the practice and evaluation of technical procedures and skills, as well as the training of teams for their performance in complex and stressful situations [4].

The most frequently performed task by a physician in the course of his/her professional life is preparing patient clinical records, also known as history taking. Getting together the relevant information such as personal and psychosocial state, related personal and family medical background, the reason for consulting, plus the physical examination findings, should allow the physician to generate a diagnosis hypothesis. The physician will confirm, reject or refine by requesting additional tests, if necessary, to generate a treatment plan [7]. This process is known as clinical reasoning, an essential skill that must be applied in every moment of patient attendance [8]. History taking and clinical reasoning are a complex process that involve interviewing skills, interpersonal skills, communication skills, knowledge elements, cognition and meta-cognition. These are the most important tools available to assist the patient. Evidence suggests that the most common medical error results from inadequate clinical reasoning [3, 9].

Simulation emerges as a new medical educational strategy allowing students to repeat a given scenario as many times as needed, make mistakes, learn from errors, ponder, receive proper feedback, trace a learning curve, and finally, improve the clinical skills required in clinical practice without compromising patients’ safety, as well as to reduce the stress produced by direct interaction with real patients [5, 10].

Virtual patient (VP) is a standardized computer software which allows simulation of real clinical scenarios that encompass the most frequent clinical cases to critical situations. The aim of VP is to expose students to virtual scenarios that would otherwise be difficult to find or to deal with in real life [11, 12]. Evidence shows that a greater number of worked cases results in a better performance in real life. VP is still under development and relatively unexplored from a pedagogic perspective. This tool has been accepted by the Liaison Committee on Medical Education (LCME) as a resource to teach rare but necessary clinical situations [13]. In addition, it is part of the United States Medical Licensing Examination (USMLE Step 3) since 1999 [13, 14].

In Colombia, Fundación Santa Fe de Bogotá (FSFB), a high complex medical institution, and SENA (National Apprenticeship Service from the acronym in Spanish) developed a computer-based simulation model i.e., *“The Virtual Patient: Simulator of Clinical Cases”*, which enables the students to prepare a clinical record, generate a diagnosis hypothesis and a treatment plan. A course was then structured under the concepts of constructivism to improve skills in history taking and clinical reasoning process for undergraduate medical students. The aim of this research was to evaluate clinical skills before and after the course, and VP performance as a pedagogic tool.

## Methods

The VP is a Web-based tool that allows professors to easily design clinical cases with teaching purposes. Every case is set up by default as a healthy patient, where the professor just needs to modify the answer to the relevant questions, and adjust it to the teaching goals. Once used, the cases can be later modified and enriched based on the acquired experience. The interaction between student and VP is established by typing questions that the software identifies by the key words contained, unfolds different and appropriate ways to ask the questions, and the answers to such questions. The tool also provides access to the physical exam findings by clicking on a human figure icon that represents the VP. Options include vital signs, sounds, videos, images, and descriptions of relevant findings. The student can also ask for additional studies such as laboratory tests and diagnostic images, propose a preliminary hypothesis and formulate a treatment. The software also has an algorithm to assess the students’ performance.

To assess the efficacy as a learning tool and the performance of VP in pedagogic practice an elective course named “Introduction to clinical reasoning” was designed and offered to undergraduate medical students without previous academic clinical experience. The course lasted 16 weeks with two-hours-per week sessions. Each session was developed around a VP clinical case of abdominal pain of different etiology, aiming to improve a specific aspect of history taking and clinical reasoning. Each case demanded to understand and handle different aspects of interview, physical examination and additional laboratory tests to achieve a diagnosis hypothesis. This design took into consideration some fundamental constructivist principles applied to education: a) Learning is a process of individual construction, which is developed by means of the activity and under the interaction with others; b) Such learning is meaningful, that is, it is anchored on the student’s prior experiences and knowledge; c) To confront repeatedly the same problem with different variations; d) Learning by doing in situations as similar as possible to the real professional environment and, e) The chance of making mistakes and to get proper permanent feedback [15–17].

The course involved 20 voluntary undergraduate medical students from first to fifth academic periods from Universidad del Rosario in the first half of 2012, who had not had a prior semiology course. Prior authorization from the University was requested, as well as an informed consent from all students involved.

To evaluate the VP as a teaching and learning tool, a quasi-experimental before-and-after study was designed. To measure the skills in history taking and clinical reasoning, considered as the learning goals of the course, a matrix was designed by the authors as experts on education and medical education [18]. Skills were defined in five descriptors: interview, physical exam, medical reasoning and relevance of additional exams and presentation of the case. Each skill was scored from 1 to 3, being 1 the lowest score (meaning that the student does not have the ability to handle the skill) and 3 being the highest score (meaning that the student handles the skill as expected). Validation of the matrix was established by three evaluators using the agreement test through the Kappa coefficient, with a 95% reliability interval. Construct and content of the matrix were validated, as well as agreement amongst the observers (Table 1).

**Table 1:** Matrix for assessment of history taking and clinical reasoning skills (Designed by authors)

Learning Goals	3	2	1
Interview	The student asks relevant and sufficient questions that reflect a logical order	The student asks some relevant and necessary questions although he/she does not follow a structured order	The student has problems with structuring relevant questions in the interview
Physical Exam	The student looks for signs in the patient and justifies such search	The student performs an incomplete physical exam or does it in a unstructured manner; does not properly recognize the case’s relevant findings	The student has no ideas on what to look for in the patient’s physical exam
Medical reasoning	The student correlates findings from the physical exam with those from the interview to approach a diagnosis	The student properly correlates some findings of the physical exam with those from the interview, or the student approaches the diagnosis, although he/she does not establish a relation that is consistent with the patient’s signs and symptoms	The student does not evidence the ability to correlate the findings from the physical exam and the interview to approach a diagnosis
Relevance of Additional Exams	The student proposes relevant strategies to acquire additional data that contribute to pinpointing the diagnosis	The student proposes some strategies that are somewhat irrelevant for the acquisition of additional data that contribute to approaching a diagnosis	The student does not propose strategies to a diagnostic approach
Presentation of case	The student presents the clinical case in a logical order and considers all the relevant information	The student presents the clinical case in a slightly unstructured manner, tries to achieve a logical order but fails to do so. Presents both relevant and irrelevant information	The student omits relevant information and does not present the clinical case coherently

To assess the learning, each student had to handle a clinical case enacted by a professional actor playing the role of a patient in the first and last course sessions. This exercise was considered as the closest to authentic clinical performance of a physician. Students’ performance was videotaped, and later assessed by three evaluators based on the matrix. The quantitative collected data was analyzed using a non-parametric analysis to two related samples, after evaluating the normality assumptions in the samples to assess the difference of the pre-course scores and the scores obtained after the 16-week course. The statistics procedures were conducted using SPSS (Statistical Packaged for Social Sciences) software version 22.

To analyze the performance of the VP tool during the course, qualitative data was collected from the students’ perceptions in a semi structured interview (focus group), professors’ perceptions (field journals), and an ethnographic work from an anthropologist who attended all sessions focusing on the interaction dynamics between students, VP and professors. The information obtained from the qualitative techniques was transcribed, digitized and coded based on the categories also defined by the authors according to the aim of the research (Table 2). For analysis and triangulation thereof, the Atlas.ti, Version. 7.0, qualitative processing tool was used.

**Table 2:** Categories for qualitative analysis of VP performance as a learning tool and its operational definitions

Analytical categories	Operational definition
Use of the tool	Understood as use and appropriation experiences of both the professor and the students in their interaction with the simulation tool
Perceptions on learning	Understood as the ways in which learnings acquired by the students in the interaction with the tool are described
Construction of knowledge and competencies	Understood as the way in which knowledge construction and development of competencies typical of medical practice are evidenced, owing to the learning environment designed
Contributions to the teaching process	Understood as those aspects that favor reflection and improvement of the teaching practice owing to the interaction with the simulation tool

The results were measured through a mixed concurrent or triangulated study [19–21].

## Results

**Fig. 1:**
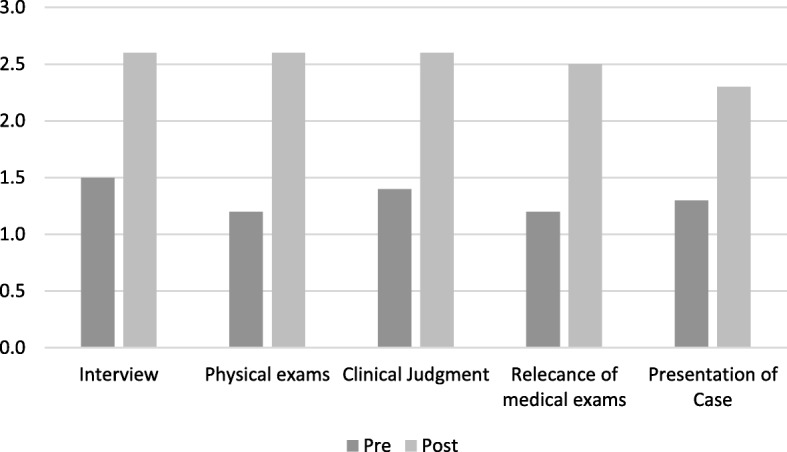
Scores pre and post. Evaluator 1

**Fig. 2:**
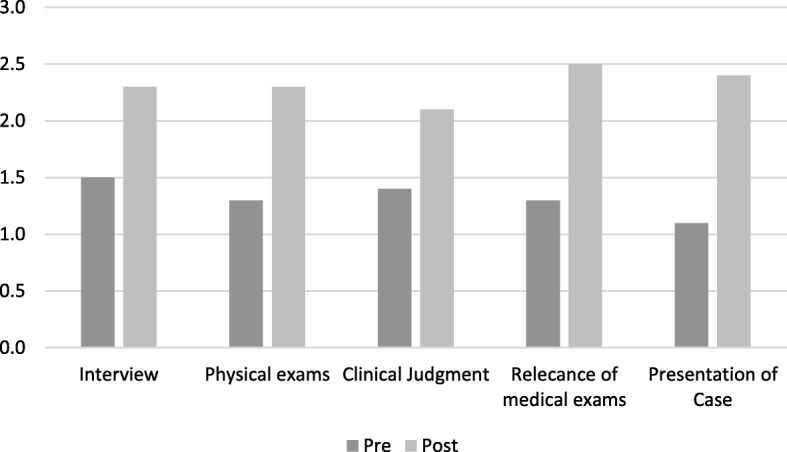
Scores pre and post. Evaluator 2

**Fig. 3:**
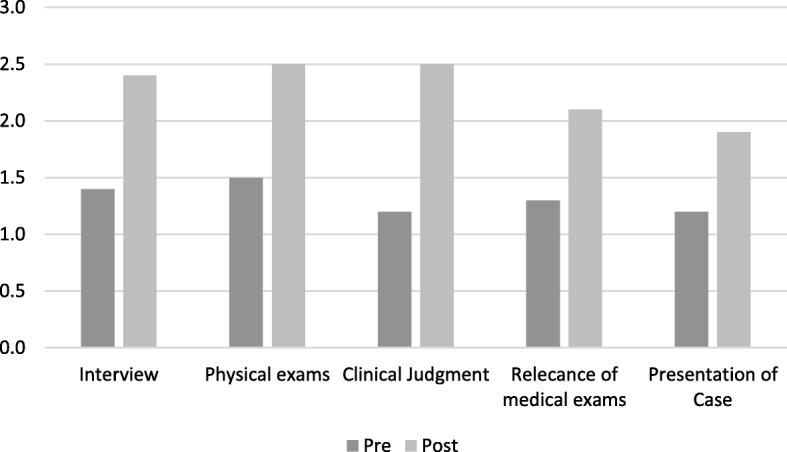
Scores pre and post. Evaluator 3

The average variation of the students’ scores between the pre-course and post-course assessment with standardized patient are presented in Figs. 1, 2 and 3 for each evaluator. Results are presented according to the evaluation matrix described above (Table 1).

The data were analyzed quantitatively to measure the difference between the scores before and after taking the course to determine the Virtual Patient’s impact on the learning process, by way of the Wilcoxon signed rank test. The student skills of history taking and clinical reasoning significantly improved after taking the course (Table 3).

**Table 3:** Wilcoxon Test Significance of the differences between the averages of the initial and final qualification assigned by each evaluator

	Evaluator 1	Evaluator 2	Evaluator 3
Interview	z = −2.37 *p* = 0.01	z = − 2.653 *p* = 0.01	z = − 2.64 *p* < 0.01
Physical exams	z = − 264 *p* < 0.01	z = − 2.42 *p* = 0.01	z = − 2.89 *p* < 0.01
Clinical judgment	z = − 2.46 *p* = 0.01	z = − 2.33 *p* = 0.02	z = − 2.74 *p* < 0.01
Relevance of medical exams	z = − 2.60 *p* < 0.01	z = − 2.46 *p* = 0.01	z = − 2.53 *p* = 0.01
Presentation of case	z = − 2.42 *p* = 0.01	z = − 2.92 *p* < 0.01	z = − 2.65 *p* < 0.01

With regard to the qualitative component, the following is a summary of findings in terms of the above-mentioned analysis categories. As can be seen, the effect from both the tool and the course design proved to be favorable for learning to take place.

### Use of the tool

Throughout the implementation of the tool during the course, it was possible to observe more advantages than disadvantages. Among the advantages of using the “Virtual Patient: Simulator of Clinical Cases” platform, one may observe that participants perceived a motivating and easy-to-use tool, which allowed for making mistakes with no consequences over the integrity of a real patient. The VP enabled getting to know a patient, tackling a clinical case, broadening and feeding the curiosity over the unknown, asking questions again, re-examining, and going back to revisit and evaluate all the information. The Table 4 describes the advantages and disadvantages drawn from the viewpoint of users.

**Table 4:** Summary of advantages and disadvantages of VP as a learning tool identified in the qualitative analysis

Advantages	Disadvantages
— Learning in the creation of logical and structured processes — More organized notion — Motivates students — Allows for making mistakes with no consequences — Integrate knowledge — There is no pressure from the professor — There is no pressure from the real patient — Intuitive use — Review and reassess the information available — Easy to use — Increases students’ curiosity — Useful early on in the career — The simulation may be repeated as many times as desired	— As the sole methodology, it becomes somewhat monotonous — Some aspects cannot be simulated; it does not replace interaction with a real patient — It does not suffice to provide for the complexity of medical doctor / patient interaction — Complementary methodologies must be used — Since it is a computer-aided simulation model, it may pose technical problems upon implementation thereof

### Perceptions on learning

It was found that the VP allowed students to learn to recognize the importance of each piece of information that may be provided by a patient, as well as the need for conducting a logical process that contributes to being more organized and methodical, and building structured mental schemes, which guide them towards possible diagnoses. By the same token, it was observed that they acquired new knowledge with the tool, they applied prior knowledge, and made connections without the pressure from the professor or from a real patient, which translated into being able to act “without fear of making a mistake.”

***Student:*** *The course on introduction to clinical reasoning has provided me with many experiences, such as: Learning how to interview a patient and how to properly conduct this interview, learning to perform an adequate physical exam, to become acquainted with the various clinical exams that must be applied in the different diseases and even suggest a convenient diagnosis; and, especially, to know what the medical act means for each one of us. For this reason, I feel I already have the foundations to face clinical procedures. The combination of capability and training with resolve and boldness allows us to continuously test new courses of action and look for bigger challenges and longer journeys.*

***Professor:*** *Well…, this case must have taught you that not everything is diagnosed by way of interviews and that the physical exam also plays a very important role … Of course, more similar cases will give you additional training on this experience, but for now I find it is all well. We will see what happens with the new case, where we will know nothing from the physical exam or from the interview, but rather from the approximation* via *clinical exams…*

### Building knowledge

One of the benefits of the VP is that it proves to be relevant for training first academic period students. Indeed, at the start of the professional career, it is useful for the student given that he/she acquires new experiences by interacting with a tool that enables the development of skills and attitudes, which are instrumental for his/her professional career.

***Observer:*** *From the Virtual Patient‘s structure, browsing the various tabs allowed the students to infer part of the clinical logic with which the doctor intervenes regarding the disease. The reading suggested as a response to concerns raised by the students on clinical logic aspects that cannot be deduced from the program’s structure or from the questions he/she asks (for instance, why does the physical exam have to be conducted in cephalocaudal direction?) exposes the same steps suggested by the program’s tabs but explaining them, defining what the physical exam is about in a detailed and orderly manner.*

***Professor:*** *Of course, as a professor you know what your student is intended to learn so activities that allow the student to build that knowledge, develop such skills, such attitudes are proposed… and few times I have been able to see that evidence of learning in the classroom as with this course… We have pushed them (they have been driven) to consider that all the information and details thereof that may be provided by a patient on his/her disease, on their complaints and reasons for consultation is of the utmost importance…; that it is important to control “your own bias” and taking the easy way out when producing a diagnosis because it may lead to serious mistakes; they have reflected on the importance of taking the medical act with all due seriousness, also given the risks involved in taking it lightly; I also believe they have seen that they cannot afford to ask questions and pay attention indefinitely, but rather, that they must start to somehow focus the interview on the relevant aspects (without neglecting the rest).*

***Student:*** *The Virtual Patient has allowed me to relate various concepts. Likewise, I feel that it has encouraged me to research those conditions that I do not understand and to improve my critical reasoning skills. I have learned to organize my ideas and I understand that all my actions must have a specific end (verify-reject ideas).*

### Contributions to the teaching process

The use of the VP tool also allowed for a better follow-up of the students’ learning process and, in the case of the professor, for a reflection on the teaching-learning process:

***Professor:*** *Once again they mentioned that the exercise has mostly taught them “everything they have yet to learn,” but in my opinion with a sense of encouragement rather than hopelessness; statements on how they became tangled up in the interview or in any finding, perhaps irrelevant (*e.g.*, a slight increase in indirect BNA), not being able to obtain something that is more relevant or useful…; statements on how they were going down a certain path and suddenly they took a different direction and stayed there…*

***Professor*** **:** *I reiterate my surprise concerning the sharpness of their deductions… I do not know exactly what it can be attributed to, but it occurred to me that the way in which we have encouraged them to be inquisitive and to search for meaningful data is working; collaborative work amongst the teams is working extremely well, because I have seen them permanently wrapped up in their discussions and with a very serious desire to approach it … Or I may be judging it too benevolently, because I am always surprised by the range of abilities of the students when they are given the freedom “to express themselves” …*

***Survey students:*** *The VP enables putting into practice and relating concepts learned, as well as strengthening concepts.*

*I feel it is a good practice, where we can not only put into practice our knowledge, but also our practice at a consultation without compromising anyone’s life or health.*

*I have felt extremely well by being able to apply what I have learned by working on the clinical cases. It is an experience that we should all have before moving forward with clinical practice.*

## Discussion

A quasi-experimental study was performed to evaluate a web-based VP as a clinical reasoning learning tool. Clinical reasoning was evaluated before and after an elective course offered to pre-clinical students in 2012 in a school of medicine in Colombia. The course was designed following constructivist principles but using the VP as the core learning tool. Students were exposed to a new and more complex clinical case in each VP session followed by small group question-guided discussions. The pre and post evaluation was conducted using standardized patients and three experts that evaluated clinical reasoning skills in each of the students.

Results attained show a remarkable contribution to the skill of students in conducting interviews and performing complete physical exams better aimed at understanding and producing a diagnosis of patient problems (*p* < 0.01). For the same reason, a contribution was made regarding guidance and the relevance for requesting additional tests geared towards confirming the diagnosis, as well as the presentation of clinical cases, a set of skills considered in this research, as evidence for the abilities of students for clinical reasoning.

It should be noted that although the VP is a Web-based simulator that uses a chat-like interviewing methodology and a click-based physical examination, history taking skills significantly improved after the course.

This finding could be related to the simulator’s function of displaying patient question suggestions to the student. One may say that this reminds the student about its relevance in the diagnostic process. In other terms, the VP proposes a logical order and familiarizes the student with the way of asking questions, previously introduced by medical instructors who apply them in their actual professional context. This results in a valuable learning experience for the student, who at first has difficulties in asking questions, especially when it comes to sensitive issues or aspects relative to the patient’s private life [22–24].

Furthermore, the analysis also revealed that the students understood the importance of exploring all possible information sources in relation to the patient’s problem, and that the logical, organized and methodical processing of data gathered helps them to propose the possible diverse diagnoses. Around the VP, students were not only able to “know” the patient and tackle the clinical cases, but also to expand and feed their curiosity over the unknown, ask questions again, re-examine, go back to revisit and evaluate all the information thus favoring a reflective learning process with a fun and easy-to-use tool, which allows them to make mistakes with no consequences over the integrity of real patients.

On another note, it is worth mentioning that the auto-filling feature of such questions is determined by the development level of the simulation model, which to a certain extent substitutes the student’s effort to consider the relevant questions within the clinical reasoning process. Nevertheless, with the recent improvements in the Natural Language Processing field of Artificial Intelligence and its applications to solving problems in medical sciences [25], this limitation could be overcome. More so, the contribution of chatbots and other subsets of artificial intelligence like machine learning to Virtual Patients and medical education opens an unexplored research area that should be addressed in the years to come.

With regard to the clinical reasoning skills, our results are similar to those previously reported in the literature by Cook and co [26]. Nonetheless, one of the most important contributions of our study relies on the qualitative analysis where we observed that students applied different resources, appropriate for their learning level, to solve problems, as suggested by Norman [8]. For this specific course, the key to reaching a diagnosis of the various cases proposed by the VP lay in a different section of the clinical record: Personal data, current disease, review by systems, medical history or physical exam. Thus, in each session they were challenged to discover different paths towards solving the diagnostic problem posed by each new VP, to resort to their memory, their expertise and comparison with prior cases. The tool allowed them to acquire new insights, apply their prior knowledge, and make connections without the pressure of the attentive presence of the professor or the real patient. Incidentally, one of the most meaningful findings from this study as it was mentioned above, is that the VP allows the students to expand and feed their curiosity over the unknown and acquire new experiences where they develop basic attitudes and skills for their professional career, which seems to be particularly useful at the start of their professional life.

We also found that the VP promoted different strategies suggested by Guraya as core for the clinical reasoning development. We observed that the VP exposed the students to.

clinical situations, activation of prior knowledge, development of case scripts, metacognition and deliberate practice, and encouraged them to prioritize differential diagnoses [27].

Nevertheless, the main limitation of the study is that the VP was immersed in a course that was designed following solid pedagogical principles to strengthen history taking and clinical reasoning skills. Hence, it is not possible to completely attribute the observed changes to the VP. However, it has been accepted that simulation facilitates learning when it is used in the appropriate conditions that include providing feedback, repetition, curriculum integration, individualized learning and controlled environment [28].

We agree with Aggarwal et al. [4] on the importance of bearing in mind that the VP and the simulation in general are merely tools for training purposes, and that success in their use does not depend so much on the accuracy of the simulation but rather on how it is used by the instructor and the student.

Given that knowledge building and the acquisition of skills are complex processes, it may not be concluded that the progress made by the students therewith is the exclusive result of the VP as a teaching tool, since it happened within a comprehensive course and other learning sources cannot be ruled out during the study’s observation period. The tool was used in a student-centered learning environment. The students relied on the professor’s guidance, who encouraged them systematically to establish connections between prior and new knowledge. There was permanent collaborative work amongst small student groups, which must have influenced the outcome.

Additionally, as has been suggested by Guraya et al., medical students prefer a more traditional approach to learning (reflectors and theorists) than a pragmatic or even an activist, and that instructional strategies should encourage students to self-directed learning [27]. However, as it has been described above, VP challenges traditional preferences but not as a single tool, rather in a constructivist context. This suggests that instructional strategies, whether technology-based or not, must include some solid principles from pedagogical theory that were incorporated to the VP course and some of what Issenberg et al. described as key for simulation-based learning [28].

Another limitation of the study was that no comparison was made with other learning strategies. The literature contains descriptions of multiple educational interventions dedicated to history taking and clinical reasoning from scripts, conferences, master lectures, demonstrations, on-line courses, small group workshops, among others used more like role playing, standardized patients, high-fidelity simulators and direct patient interviews. Moreover, it is possible to find systematic reviews that include studies where VP has been compared with some of those strategies and where it showed positive results against traditional lectures and even standardized patients, but evidence is not [7] conclusive and heterogeneity of the data suggest that more research is needed [7, 26, 29]. The remaining question whether VP is superior to other strategies could be secondary because the qualitative analysis allows establishing that the tool was unable to replace other teaching strategies completely. In fact, despite the participants exhibiting great interest and enthusiasm for the work with the VP, within the context of the full course, the need for using other pedagogic and evaluation tools (videos, mock patients, visits from advanced peers) was evidenced, since the current degree of development of the VP is insufficient to simulate the complexity of the physician-patient interaction, the core act of the clinical activity and main settings for medicine.

From a cost perspective, the cost per hour of the VP was USD 12, which is noteworthy as it is low compared to high-fidelity simulators (between USD 160 and 800 per hour) and standardized patients (from USD 30 to 40 per hour). The cost of the software and faculty time was similar to what was previously reported in the literature [30]: $49,000. USD. Nonetheless, the scope of the study did not include a cost-effectiveness analysis and prospective studies are needed.

## Conclusions

We conclude that the VP as a learning tool showed a significant contribution to improving history taking and clinical reasoning skills in students of medicine in a preclinical environment. However, VP was used as the main tool of a course that was designed with solid pedagogical foundation, making it difficult to completely attribute the results to the single tool.

Results suggest that a learning environment that promotes individual construction through peer interaction, meaningful learning, repetition of the same problem with different variations, learning by doing and a proper permanent feedback are essential to ensuring the efficacy of a Web-based virtual patient for developing clinical reasoning and history taking skills. However, controlled trials that compare the VP as part of a more traditional course versus a constructivist environment are needed to confirm our findings.

## Abbreviations

LCME: Liaison Committee on Medical Education
SENA: National Training service [Servicio Nacional de Aprendizaje]
USMLE: Unites States Medical Licensing Examination
VP: Virtual Patient
